# (*E*)-5-Meth­oxy-2-(*o*-tolyl­imino­meth­yl)phenol

**DOI:** 10.1107/S1600536809055615

**Published:** 2010-01-09

**Authors:** Çiğdem Albayrak, Başak Koşar, Arzu Özek, Mustafa Odabaşoğlu, Orhan Büyükgüngör

**Affiliations:** a Department of Science Education, Sinop University, TR-57100 Sinop, Turkey; b Department of Physics, Ondokuz Mayıs University, TR-55139 Samsun, Turkey; c Chemistry Programme, Pamukkale University, TR-20159 Denizli, Turkey

## Abstract

In the title compound, C_15_H_15_NO_2_, the phenol group make dihedral angles of 2.4 (2) and 24.1 (9)° with the imine linkage (–C=N–) and the phenyl group, respectively, and the mol­ecule adopts the enol–imine tautomeric form, so the mol­ecular structure is stabilized by a strong intra­molecular O—H⋯N hydrogen bond. The crystal structure features a weak C—H⋯π inter­action.

## Related literature

For the relationships between thermochromism and photochromism and the planarity of mol­ecules, see: Moustakali-Mavridis *et al.* (1980 ▶). For bond lengths in related structures, see: Tanak & Yavuz (2009 ▶); Koşar *et al.* (2009 ▶.
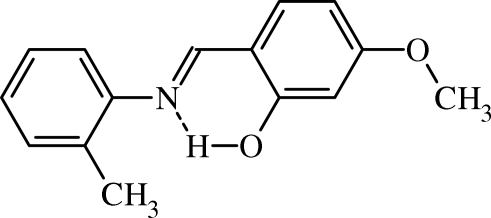


## Experimental

### 

#### Crystal data

C_15_H_15_NO_2_
*M* *_r_* = 241.28
Monoclinic, 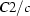

*a* = 22.3720 (16) Å
*b* = 7.3191 (4) Å
*c* = 22.1704 (14) Å
β = 136.094 (4)°
*V* = 2517.5 (3) Å^3^
*Z* = 8
Mo *K*α radiation
μ = 0.09 mm^−1^
*T* = 293 K
0.80 × 0.46 × 0.21 mm

#### Data collection

Stoe IPDS II diffractometer
Absorption correction: integration (*X-RED*; Stoe & Cie, 2002 ▶) *T*_min_ = 0.948, *T*_max_ = 0.984
17732 measured reflections
2914 independent reflections
1935 reflections with *I* > 2σ(*I*)
*R*_int_ = 0.053

#### Refinement

*R*[*F* ^2^ > 2σ(*F* ^2^)] = 0.049
*wR*(*F* ^2^) = 0.122
*S* = 1.03
2914 reflections
167 parameters
H atoms treated by a mixture of independent and constrained refinement
Δρ_max_ = 0.13 e Å^−3^
Δρ_min_ = −0.12 e Å^−3^

### 

Data collection: *X-AREA* (Stoe & Cie, 2002 ▶); cell refinement: *X-AREA*; data reduction: *X-RED* (Stoe & Cie, 2002 ▶); program(s) used to solve structure: *SHELXS97* (Sheldrick, 2008 ▶); program(s) used to refine structure: *SHELXL97* (Sheldrick, 2008 ▶); molecular graphics: *ORTEP-3 for Windows* (Farrugia, 1997 ▶); software used to prepare material for publication: *WinGX* (Farrugia, 1999 ▶).

## Supplementary Material

Crystal structure: contains datablocks global, I. DOI: 10.1107/S1600536809055615/bx2258sup1.cif

Structure factors: contains datablocks I. DOI: 10.1107/S1600536809055615/bx2258Isup2.hkl

Additional supplementary materials:  crystallographic information; 3D view; checkCIF report

## Figures and Tables

**Table 1:** Hydrogen-bond geometry (Å, °) *Cg* is the centroid of the C1–C6 ring.

*D*—H⋯*A*	*D*—H	H⋯*A*	*D*⋯*A*	*D*—H⋯*A*
O1—H16⋯N1	0.95 (2)	1.75 (2)	2.5992 (19)	148.3 (19)
C15—H15*B*⋯*Cg*^i^	0.96	2.98	3.900 (2)	160

Symmetry code: (i) 
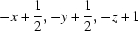
.

**Fig. 1.**
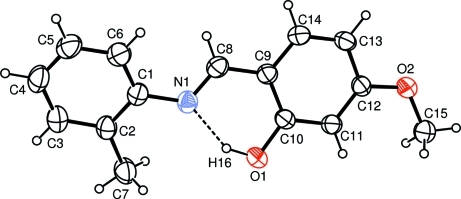
Thermal ellipsoid view of the title compound. Displacement ellipsoids are drawn at the 30% probability level and H atoms are shown as small spheres with arbitrary radii. Dashed line indicates the intramolecular hydrogen bond.

**Fig. 2.**
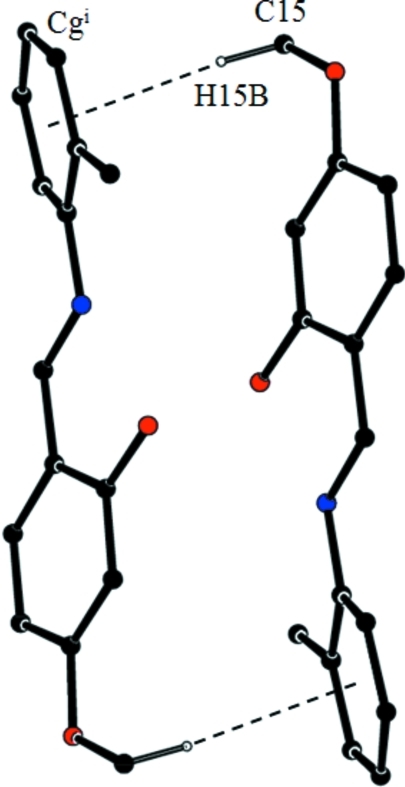
Part of crystal structure of molecule, showing the C—H···π bonds. For clarity, H atoms not included in intermolecular bonding have been omitted. For symmetry codes, see Table 1.

## supplementary crystallographic information

### Comment

Schiff bases are formed by reaction of a primary amine and an aldehyde and have a wide area of usage as ligands in coordination chemistry. Especially *o*-hydroxy Schiff base derivatives are important classes have attracted the interest of chemists and physicist because of their photochromic and thermochromic features in the solid state. These features are caused by the proton transfer to N atom from O atom with light in photochromic or with temperature in thermochromic Schiff bases. It has been claimed that the molecules showing thermochromism are planar and showing photochromism are non-planar (Moustakali-Mavridis *et al.*, 1980). In general, *o*-Hydroxy Schiff bases can be found at two possible tautomeric forms called as phenol-imine and keto-amine. The molecular structure of the title compound (I), is the enol-imine tautomer, as indicated by the following bond lengths: N1═C8 (1.284 (2) Å), C8—C9 (1.439 (2) Å) and C10—O1 (1.3445 (18) Å). These bond lengths are in a good agreement with observed for (*E*)-2-[(4-Chlorophenyl)iminomethyl]-5- methoxyphenol [1.282 (2), 1.436 (2) and 1.3452 (18) Å; Koşar *et al.*, 2009], which is also enol-imine tautomer. The same bond lengths are comparable with observed for (*E*)-2-[(2-Hydroxy-5-nitrophenyl)-iminomethyl]-4-nitrophenolate [1.288, 1.420 and 1.2749 Å; Tanak & Yavuz, 2009], which is a keto-amine tautomer. The molecule is not planar  and make a dihedral angle of 2.4 (2) and 24.1 (9)° with the imine linkage and the phenyl group respectively and shows photochromic features. As a result of enol-imine form of the molecule, there is a strong intramolecular hydrogen bond between the atom O1 and atom N1 (Fig. 1).The crystal structure is primarily determined by one weak C—H···π ( Cg = C1/C6) and van der Waals interactions, Table 1.

### Experimental

For the preparation of (*E*)-5-methoxy-2-[(*o*-tolylimino)methyl]phenol compound the mixture of 4-methoxysalicylaldehyde (0.5 g, 3.3 mmol) in ethanol (20 ml) and 2-methylaniline (0.35 g, 3.3 mmol) in ethanol (20 ml) was stirred for 1 h under reflux. The crystals suitable for X-ray analysis were obtained from ethanol by slow evaporation (yield; %76, m.p.; 372 K).

### Refinement

All H atoms except for H16 were refined using riding model with C—H distances of 0.96 Å for methyl group and 0.93 Å for aromatic groups. The displacement parameters of these H atoms were fixed at 1.2 *U*_eq_ of their parent carbon atom for aromatic groups and 1.5 *U*_eq_ of their parent atoms for methyl group.

### Crystal data

**Table:**

C_15_H_15_NO_2_	*F*(000) = 1024
*M**_r_* = 241.28	*D*_x_ = 1.273 Mg m^−^^3^
Monoclinic, *C*2/*c*	Melting point: 372 K
Hall symbol: -C 2yc	Mo *K*α radiation, λ = 0.71073 Å
*a* = 22.3720 (16) Å	Cell parameters from 2073 reflections
*b* = 7.3191 (4) Å	θ = 1.9–28.0°
*c* = 22.1704 (14) Å	µ = 0.09 mm^−^^1^
β = 136.094 (4)°	*T* = 293 K
*V* = 2517.5 (3) Å^3^	Prism, yellow
*Z* = 8	0.80 × 0.46 × 0.21 mm

### Data collection

**Table:**

Stoe IPDS II diffractometer	2914 independent reflections
Radiation source: fine-focus sealed tube	1935 reflections with *I* > 2σ(*I*)
graphite	*R*_int_ = 0.053
Detector resolution: 6.67 pixels mm^-1^	θ_max_ = 27.5°, θ_min_ = 2.0°
ω scan	*h* = −28→28
Absorption correction: integration (*X-RED*; Stoe & Cie, 2002)	*k* = −9→9
*T*_min_ = 0.948, *T*_max_ = 0.984	*l* = −28→28
17732 measured reflections	

### Refinement

**Table:**

Refinement on *F*^2^	Primary atom site location: structure-invariant direct methods
Least-squares matrix: full	Secondary atom site location: difference Fourier map
*R*[*F*^2^ > 2σ(*F*^2^)] = 0.049	Hydrogen site location: inferred from neighbouring sites
*wR*(*F*^2^) = 0.122	H atoms treated by a mixture of independent and constrained refinement
*S* = 1.03	*w* = 1/[σ^2^(*F*_o_^2^) + (0.0534*P*)^2^ + 0.4484*P*] where *P* = (*F*_o_^2^ + 2*F*_c_^2^)/3
2914 reflections	(Δ/σ)_max_ < 0.001
167 parameters	Δρ_max_ = 0.13 e Å^−^^3^
0 restraints	Δρ_min_ = −0.12 e Å^−^^3^

### Special details

**Table:**

Geometry. All e.s.d.'s (except the e.s.d. in the dihedral angle between two l.s. planes) are estimated using the full covariance matrix. The cell e.s.d.'s are taken into account individually in the estimation of e.s.d.'s in distances, angles and torsion angles; correlations between e.s.d.'s in cell parameters are only used when they are defined by crystal symmetry. An approximate (isotropic) treatment of cell e.s.d.'s is used for estimating e.s.d.'s involving l.s. planes.
Refinement. Refinement of *F*^2^ against ALL reflections. The weighted *R*-factor *wR* and goodness of fit *S* are based on *F*^2^, conventional *R*-factors *R* are based on *F*, with *F* set to zero for negative *F*^2^. The threshold expression of *F*^2^ > σ(*F*^2^) is used only for calculating *R*-factors(gt) *etc*. and is not relevant to the choice of reflections for refinement. *R*-factors based on *F*^2^ are statistically about twice as large as those based on *F*, and *R*- factors based on ALL data will be even larger.

### Fractional atomic coordinates and isotropic or equivalent isotropic displacement parameters (Å^2^)

**Table:**

	*x*	*y*	*z*	*U*_iso_*/*U*_eq_	
C1	0.44408 (9)	0.4751 (2)	0.61857 (10)	0.0585 (4)	
C2	0.48769 (10)	0.4298 (2)	0.59731 (11)	0.0628 (4)	
C3	0.54314 (12)	0.5583 (3)	0.61274 (13)	0.0750 (5)	
H3	0.5734	0.5293	0.5998	0.090*	
C4	0.55431 (13)	0.7276 (3)	0.64672 (13)	0.0829 (6)	
H4	0.5932	0.8101	0.6584	0.100*	
C5	0.50793 (15)	0.7743 (3)	0.66328 (13)	0.0839 (6)	
H5	0.5141	0.8900	0.6846	0.101*	
C6	0.45217 (12)	0.6501 (2)	0.64842 (11)	0.0708 (5)	
H6	0.4198	0.6833	0.6584	0.085*	
C7	0.47509 (15)	0.2476 (3)	0.55859 (15)	0.0866 (6)	
H7A	0.4156	0.2332	0.5044	0.104*	
H7B	0.4913	0.1524	0.5979	0.104*	
H7C	0.5103	0.2405	0.5486	0.104*	
C8	0.37691 (10)	0.3374 (2)	0.65349 (10)	0.0626 (4)	
H8	0.3972	0.4336	0.6917	0.075*	
C9	0.32840 (9)	0.1941 (2)	0.64692 (9)	0.0556 (4)	
C10	0.29831 (9)	0.0417 (2)	0.59225 (10)	0.0543 (4)	
C11	0.25453 (10)	−0.0984 (2)	0.58927 (10)	0.0568 (4)	
H11	0.2359	−0.1996	0.5539	0.068*	
C12	0.23880 (9)	−0.0871 (2)	0.63893 (10)	0.0555 (4)	
C13	0.26662 (11)	0.0640 (2)	0.69232 (10)	0.0624 (4)	
H13	0.2552	0.0717	0.7251	0.075*	
C14	0.31064 (10)	0.2001 (2)	0.69596 (10)	0.0630 (4)	
H14	0.3295	0.2999	0.7320	0.076*	
C15	0.16589 (13)	−0.3735 (3)	0.58692 (13)	0.0782 (5)	
H15A	0.2134	−0.4352	0.6026	0.094*	
H15B	0.1254	−0.3358	0.5272	0.094*	
H15C	0.1381	−0.4547	0.5946	0.094*	
N1	0.39313 (8)	0.33716 (19)	0.60847 (8)	0.0602 (4)	
O1	0.31237 (8)	0.02673 (19)	0.54283 (8)	0.0683 (3)	
O2	0.19682 (8)	−0.21732 (17)	0.64089 (8)	0.0701 (3)	
H16	0.3415 (14)	0.134 (3)	0.5514 (14)	0.102 (7)*	

### Atomic displacement parameters (Å^2^)

**Table:**

	*U*^11^	*U*^22^	*U*^33^	*U*^12^	*U*^13^	*U*^23^
C1	0.0516 (8)	0.0595 (10)	0.0495 (8)	−0.0007 (7)	0.0314 (7)	0.0071 (7)
C2	0.0593 (9)	0.0617 (10)	0.0615 (9)	0.0016 (8)	0.0415 (8)	0.0104 (8)
C3	0.0684 (10)	0.0764 (12)	0.0796 (12)	−0.0016 (9)	0.0531 (10)	0.0124 (10)
C4	0.0808 (12)	0.0762 (14)	0.0748 (12)	−0.0196 (10)	0.0504 (11)	0.0049 (10)
C5	0.1089 (15)	0.0595 (11)	0.0715 (12)	−0.0139 (11)	0.0611 (12)	−0.0009 (9)
C6	0.0797 (11)	0.0627 (11)	0.0640 (10)	0.0004 (9)	0.0498 (10)	0.0056 (8)
C7	0.1093 (15)	0.0689 (12)	0.1185 (17)	−0.0058 (11)	0.0944 (15)	−0.0008 (12)
C8	0.0550 (9)	0.0661 (10)	0.0533 (9)	−0.0033 (7)	0.0346 (8)	−0.0030 (8)
C9	0.0513 (8)	0.0622 (9)	0.0488 (8)	−0.0004 (7)	0.0345 (7)	−0.0003 (7)
C10	0.0511 (8)	0.0643 (10)	0.0499 (8)	0.0035 (7)	0.0371 (7)	0.0020 (7)
C11	0.0560 (8)	0.0620 (10)	0.0525 (8)	−0.0011 (7)	0.0390 (7)	−0.0040 (7)
C12	0.0517 (8)	0.0647 (10)	0.0515 (8)	0.0001 (7)	0.0377 (7)	0.0026 (7)
C13	0.0675 (9)	0.0751 (11)	0.0551 (9)	−0.0014 (9)	0.0476 (8)	−0.0041 (8)
C14	0.0649 (9)	0.0677 (10)	0.0542 (9)	−0.0054 (8)	0.0422 (8)	−0.0093 (8)
C15	0.0935 (13)	0.0710 (12)	0.0880 (13)	−0.0158 (10)	0.0713 (12)	−0.0092 (10)
N1	0.0544 (7)	0.0645 (9)	0.0573 (8)	−0.0016 (6)	0.0387 (7)	0.0034 (7)
O1	0.0801 (8)	0.0751 (8)	0.0744 (8)	−0.0093 (7)	0.0640 (7)	−0.0089 (6)
O2	0.0831 (8)	0.0741 (8)	0.0732 (7)	−0.0146 (6)	0.0630 (7)	−0.0096 (6)

### Geometric parameters (Å, °)

**Table:**

C1—C6	1.392 (2)	C8—H8	0.9300
C1—C2	1.395 (2)	C9—C14	1.402 (2)
C1—N1	1.415 (2)	C9—C10	1.411 (2)
C2—C3	1.389 (2)	C10—O1	1.3445 (18)
C2—C7	1.499 (3)	C10—C11	1.387 (2)
C3—C4	1.376 (3)	C11—C12	1.379 (2)
C3—H3	0.9300	C11—H11	0.9300
C4—C5	1.370 (3)	C12—O2	1.3604 (18)
C4—H4	0.9300	C12—C13	1.397 (2)
C5—C6	1.378 (3)	C13—C14	1.361 (2)
C5—H5	0.9300	C13—H13	0.9300
C6—H6	0.9300	C14—H14	0.9300
C7—H7A	0.9600	C15—O2	1.423 (2)
C7—H7B	0.9600	C15—H15A	0.9600
C7—H7C	0.9600	C15—H15B	0.9600
C8—N1	1.284 (2)	C15—H15C	0.9600
C8—C9	1.439 (2)	O1—H16	0.95 (2)
			
C6—C1—C2	119.74 (16)	C14—C9—C10	117.70 (15)
C6—C1—N1	123.08 (16)	C14—C9—C8	120.87 (15)
C2—C1—N1	117.18 (15)	C10—C9—C8	121.42 (14)
C3—C2—C1	118.29 (17)	O1—C10—C11	118.22 (15)
C3—C2—C7	120.63 (17)	O1—C10—C9	121.17 (15)
C1—C2—C7	121.08 (15)	C11—C10—C9	120.60 (14)
C4—C3—C2	121.47 (19)	C12—C11—C10	119.71 (15)
C4—C3—H3	119.3	C12—C11—H11	120.1
C2—C3—H3	119.3	C10—C11—H11	120.1
C5—C4—C3	119.85 (18)	O2—C12—C11	124.24 (15)
C5—C4—H4	120.1	O2—C12—C13	115.10 (14)
C3—C4—H4	120.1	C11—C12—C13	120.65 (15)
C4—C5—C6	120.1 (2)	C14—C13—C12	119.43 (15)
C4—C5—H5	120.0	C14—C13—H13	120.3
C6—C5—H5	120.0	C12—C13—H13	120.3
C5—C6—C1	120.39 (19)	C13—C14—C9	121.88 (16)
C5—C6—H6	119.8	C13—C14—H14	119.1
C1—C6—H6	119.8	C9—C14—H14	119.1
C2—C7—H7A	109.5	O2—C15—H15A	109.5
C2—C7—H7B	109.5	O2—C15—H15B	109.5
H7A—C7—H7B	109.5	H15A—C15—H15B	109.5
C2—C7—H7C	109.5	O2—C15—H15C	109.5
H7A—C7—H7C	109.5	H15A—C15—H15C	109.5
H7B—C7—H7C	109.5	H15B—C15—H15C	109.5
N1—C8—C9	122.35 (16)	C8—N1—C1	121.51 (15)
N1—C8—H8	118.8	C10—O1—H16	107.6 (13)
C9—C8—H8	118.8	C12—O2—C15	117.67 (13)
			
C6—C1—C2—C3	4.6 (2)	C8—C9—C10—C11	177.51 (14)
N1—C1—C2—C3	−175.30 (14)	O1—C10—C11—C12	−179.80 (14)
C6—C1—C2—C7	−175.26 (16)	C9—C10—C11—C12	1.3 (2)
N1—C1—C2—C7	4.9 (2)	C10—C11—C12—O2	−179.71 (14)
C1—C2—C3—C4	−1.1 (3)	C10—C11—C12—C13	−0.1 (2)
C7—C2—C3—C4	178.75 (18)	O2—C12—C13—C14	178.83 (15)
C2—C3—C4—C5	−2.2 (3)	C11—C12—C13—C14	−0.8 (2)
C3—C4—C5—C6	2.0 (3)	C12—C13—C14—C9	0.6 (2)
C4—C5—C6—C1	1.6 (3)	C10—C9—C14—C13	0.6 (2)
C2—C1—C6—C5	−4.9 (2)	C8—C9—C14—C13	−178.44 (15)
N1—C1—C6—C5	174.98 (15)	C9—C8—N1—C1	−177.22 (14)
N1—C8—C9—C14	−178.93 (15)	C6—C1—N1—C8	−25.8 (2)
N1—C8—C9—C10	2.1 (2)	C2—C1—N1—C8	154.04 (15)
C14—C9—C10—O1	179.60 (14)	C11—C12—O2—C15	−1.2 (2)
C8—C9—C10—O1	−1.4 (2)	C13—C12—O2—C15	179.16 (15)
C14—C9—C10—C11	−1.5 (2)		

### Hydrogen-bond geometry (Å, °)

**Table:**

Cg is the centroid of the C1–C6 ring.

**Table:**

*D*—H···*A*	*D*—H	H···*A*	*D*···*A*	*D*—H···*A*
O1—H16···N1	0.95 (2)	1.75 (2)	2.5992 (19)	148.3 (19)
C15—H15B···Cg^i^	0.96	2.98	3.900 (2)	160

Symmetry codes: (i) −*x*+1/2, −*y*+1/2, −*z*+1.
